# Ten simple rules towards healthier research labs

**DOI:** 10.1371/journal.pcbi.1006914

**Published:** 2019-04-11

**Authors:** Fernando T. Maestre

**Affiliations:** Departamento de Biología y Geología, Física y Química Inorgánica, Universidad Rey Juan Carlos, Móstoles, Spain; Whitehead Institute for Biomedical Research, UNITED STATES

## Abstract

The negative effects of extremely competitive academic and research environments on the performance and health of researchers are well known and common worldwide. The prevalence of these effects, particularly among early career researchers, calls for a more humane and people-centered way of working within research labs. Although there is growing concern about the urgent need for a better life–work balance when doing science, there are not many examples about how this could be achieved in practice. In this article, I introduce 10 simple rules to make the working environment of research labs more nurturing, collaborative, and people-centered. These rules are directed towards existing and future principal investigators (PIs) but will be of interest to anyone working in a research lab and/or dealing with how to improve working conditions for scientists.

“We are all smart. Distinguish yourself by being kind.”—Charles Gordon

## Introduction

Doing science often looks like a dream job, but many aspects of current scientific practice across the world make it a stressful activity. These include the shortage of scientific positions, the fear of being scooped by competing labs, the pressure to publish in high profile journals −which are both a key indicator of success and prestige and crucial to secure positions/promotions−the uncertainty imposed by short-term contracts, and even the competition with other lab mates [1–5]. Scientists must also confront established practices that (i) force them to become workaholics if they want to get a permanent position and/or become successful [6], (ii) often promote harassment against women and minorities [7, 8], and (iii) emphasize success when failures (of experiments and/or simulations) and rejections (of articles, proposals, and job applications) are inseparable from the scientific endeavour [9, 10]. In addition, evaluation systems in place in many countries reward the number of publications in indexed journals over their quality [11–14], further pushing scientists to publish as much and as quickly as possible. This adds additional stress to researchers and promotes scientific malpractices such as nepotism and collusion, which negatively affect their well-being [11, 14–16]. With these antecedents, it is not surprising to read that many young and talented principal investigators (PIs) are frustrated [1] or to see surveys revealing that over 14% of non-PIs choose terms such as “competitive, stressful, or toxic” to describe their labs [17]. Even more worrying, recent studies and surveys have revealed that mental problems in academia are on the rise, with graduate students showing alarming rates of anxiety and depression [18, 19].

The issues described above call for a shift in scientific practice, which is urgently needed to protect scientists against health risks and abuses associated with extreme pressure and other negative habits that have plagued research labs for decades. Such a shift would also benefit the entire scientific enterprise by favoring more human and creative environments in which ideas can flourish and ground-breaking discoveries and innovations can be made, something that is at odds with the pressure to publish quickly and often [11, 15, 20]. Although there is growing concern about the urgent need for a better life–work balance and for creating healthier working habits [17–19], there are few examples of how this could be achieved in practice [e.g., 21–25]. Indeed, limited information and resources on this topic and lack of training on management and/or leadership skills by PIs are often invoked as barriers to effectively create healthier working environments in academic and research institutions [17, 25].

In this article, I present 10 simple rules based on my experience as a PI to make research labs nurturing, collaborative, and people-centered research environments. As leaders of research labs and role models for early career scientists, PIs, and particularly those that are well-established in their respective fields, have a critical role to play in promoting a shift towards creating healthier research environments. Therefore, these rules are directed toward existing and future PIs but will be of interest to anyone working in a research lab and/or dealing with how to improve working conditions for scientists.

## Rule 1: Promote the well-being of your lab members

We work more efficiently and are more creatively when we are happy. This is well known by psychologists studying productivity in the workplace across a wide range of jobs [e.g., 26–28]. The well-being of lab members must be a priority for PIs, who should devote important efforts to make research labs places where everyone can work in the best conditions possible while at the same time enjoying doing science. There is no unique path to achieve this but putting yourself in the situation of the others; being kind; banning all forms of harassment and discrimination within the lab; being sensitive when it comes to dealing with personal, family and health situations; and carefully listening to lab members regarding any matter related to their work can substantially improve the well-being of lab members. It is important to let your lab members know that you care about them and that you are here to listen to and to help them to overcome any issues that may negatively affect their work.

## Rule 2: Let people set their own schedules

As PIs, we should not strictly control lab members’ schedules, and we should be flexible regarding their working preferences. Some people prefer to come early in the morning to have the afternoons free, whereas others prefer to do the opposite. Sometimes, it is more effective to stay at home when analyzing data and writing or to reconcile work and family obligations. PIs should facilitate these arrangements, because scientists should be evaluated by the outcome of their work rather than by the time they spend in their workplace (which for many researchers can be any spot with a computer and an internet connection). As Gandalf the Grey says, “All we have to decide is what to do with the time that is given us” [29], and it is the responsibility of lab members to use their working time wisely and to do whatever best works for them. Of course, when applying this rule, we must also keep in mind both differences among disciplines and the particular challenges faced by each lab. For example, graduate students and postdocs can work on an individual programming project or on the writing of a manuscript at home if they are more productive, but this may not work in collaborative projects requiring teammates to coordinate schedules.

From my experience, offering lab members this flexibility works very well for most people; it also helps graduate students and postdocs to learn how to manage their time effectively, something very important given the multiple tasks they will have to do when and if they become PIs. It must also be noted that offering lab members flexibility to set their own schedules does not remove our obligations as PIs to properly supervise them. We must hold periodic meetings with lab members to check the progress of their work. This is also very important to let them know that we care and are on top of what they do, as well as to discuss solutions when problems arise or when expectations are not met. Not doing so is indeed a source of frustration, particularly for graduate students [17].

## Rule 3: Gratitude is the sign of noble souls

Psychologists are well aware of the multiple benefits of being grateful [30, 31]. This not only has very positive knock-on effects on the work and personal well-being of lab members but also helps to build confidence and compromise among them. Showing our gratitude to lab members is important because their work, from the accounting done by administrative assistants to the data gathered by technicians or the writing of manuscripts by graduate students or postdocs, is crucial to ensure the smooth running of a research group. PIs can also show lab members how important their work is by providing rapid feedback to their requests, questions, and manuscript drafts. This is something that they really appreciate, particularly graduate students and postdocs, and contributes to boosting their motivation. Although at particularly busy periods it may not be possible to provide quick feedback to the request of lab members, trying to do so should always be our priority as PIs.

## Rule 4: Treat your lab members as your teammates

It is not uncommon to find labs with clearly established hierarchies and “top-down” approaches, particularly when it comes to the treatment of graduate students and technicians. Such an approach promotes toxic relationships and limits the capacity of lab members to think critically. As PIs, we must have the vision, set the research priorities for our labs, and have the last say on multiple matters. However, treating lab members as mere executors of our instructions rather than as colleagues that have an informed opinion about the work they do (and hence about how to improve it!) is a huge lost opportunity. We must listen to and take the opinion and advice of technicians, graduate students, and postdocs very seriously and often discuss with them ideas for projects and papers, lab procedures, and day-to-day issues affecting their work and well-being.

For this rule to work, PIs must also learn to delegate important work. Doing so relieves PIs of extra duties that other lab members can do more efficiently, such as doing chemical analyses on the lab or filling administrative forms. It also motivates lab members to become more engaged with lab projects and overall research objectives, thus contributing to teambuilding.

## Rule 5: Create a collaborative environment within your lab

Collaboration is a cornerstone of current scientific practice [32] that allows scientists to tackle ambitious, expensive, or multidisciplinary projects not amenable to a single lab. Doing science as a collective endeavour also brings multiple opportunities for learning and professional development, particularly for early career researchers. Therefore, as PIs, we must actively practice and foster collaborations within our labs, which also helps lab members to get along better with each other (something very important to maintain a happy and productive lab!). These collaborations also help to foster long-term relationships that can also be very fruitful for their professional development. Within-lab collaborations can be nurtured by setting up common lab projects, encouraging meetings and discussions involving all lab members, providing time and resources to develop side projects and/or ideas coming from them, conducting retreats and regular meetings outside the lab, and facilitating interactions between graduate students and postdocs. Establishing priorities and identifying needs in advance, knowing how to organize the work of everyone, and being gentle in the way we ask for help when needed also contribute to setting up effective collaborations within our labs.

Creating a collaborative, rather than competitive, environment within research labs not only helps everyone pull in the same direction but also fosters the motivation, productivity, and creativity of lab members. This also prepares them to set up collaborations with colleagues from other institutions, which are also very important for their career development.

## Rule 6: Remember that every lab member is unique

A key rule we must follow as PIs is not to compare our lab members to one another or with ourselves when we were students and/or postdocs. Comparing lab members will often result in increased stress and/or anxiety levels, reducing their performance and capabilities. Every person is different, and, as PIs, we should never forget that our major role as mentors is to foster everyone’s capabilities and help them to fulfill their potential and professional ambitions. Therefore, we should make every effort to identify these goals and to support them by choosing appropriate projects and forging the right contacts. We must keep in mind that our objective as PIs is to help our lab members reach as far as they can and/or want, not as far as we want.

## Rule 7: Respect working hours, public holidays, and vacations

Working rules commonly in place in labs around the world often mean that academics work all day long, on weekends, and even during holidays [1, 6]. The stress associated with this excessive work without a life outside the lab is one of the main reasons behind the increase in mental problems in academia, particularly among early career researchers and young PIs [1, 19]. This also has many other deleterious effects on the health and well-being of researchers (see [33] for a review). Therefore, PIs should not expect lab members to work beyond normal hours, during weekends, and on holidays. We all face moments (e.g., deadlines for grant submissions, setup of large experiments, field campaigns) in which we must work hard. But this should be the exception, not the rule. Doing so is unsustainable in the long-term and contributes to generating expectations about the research environment that are neither realistic for many people nor desirable and/or healthy for the whole scientific community.

This rule can be seen as contradictory by junior PIs or those who are running labs that are short of labor and other resources, who are struggling with keeping a lab funded, or who are worried about tenure or establishing their reputation. We must also keep in mind the large differences that exist between different countries and cultures about what constitutes a “normal” working week, the length of annual holidays, and the pressures induced by the requirements to getting a job or being promoted. But even in these cases, it is important to remember that our working conditions are regulated by law and our contracts, and that working for long hours is not a sine qua non condition for being successful as a scientist (something that is intimately linked to our personal life [35]), as multiple examples from around the world illustrate [6, 24, 34].

Despite the importance of this rule for maintaining healthier research labs, as PIs we should also respect those lab members who choose to work for long hours because they feel that they must do so to be more productive, to secure a position in science, or because they have the ambition or the desire to be so. In the end, this is part of their freedom and autonomy (things are seen very differently from a permanent and/or well-established position) and we cannot forget that scientific productivity is important for the future career prospects of PhD students and postdocs. But at the same time, we should discourage these habits and advise them about the long-term ill effects that they may have on their health and well-being.

To gain the maximum benefit from this rule, PIs must also openly discuss and share with all lab members resources and experiences and/or tips to work more efficiently so they can maximize their productivity within normal working hours to avoid the need to work beyond them.

## Rule 8: Give credit where credit is due

We all have either experienced or heard about PIs who dictate authorship inclusion or order, or who insist on being authors on every paper produced by lab members, regardless of their contribution. This practice only benefits those in power, discourages effective collaborations, impedes the productivity and creativity of lab members, and fosters frustration and distrust among non-PIs. Therefore, it should be abolished. As PIs, we must openly discuss coauthorship issues with our lab members and train them on the importance of carefully evaluating the merits of coauthors before submitting publications. Failing to include meritorious coauthors or including undeserving coauthors can easily lead to frustrations and misunderstandings that must be avoided.

There are multiple ways we can give proper credit to PIs, including involving technicians in publications when they have contributed to them, leaving “senior” (e.g., last author) positions to postdocs when they had the idea of the study and are not first authors, declining authorship in articles in which we did not participate, and acknowledging in talks with colleagues, seminars, and scientific meetings the intellectual authorship of publications or ideas coming from our lab members.

## Rule 9: Destigmatize failure and celebrate success

Active scientists face rejection of their papers, grants, and job applications continuously, no matter what their career stage and status are [9, 10, 36]. Focusing on success while living under continuous rejection may put more pressure on the work of our graduate students and postdocs, increasing their frustration and anxiety levels when their articles or applications are rejected. And although rejection always hurts, scientists must embrace it as another (and important) part of their job [9, 10]. Initiatives to normalize rejection include the building of “a CV of failures” (see [37] for a great example), talking openly and sharing our experiences about rejection, and discussing with lab members the potential reasons for a particular rejection and how to avoid it the next time. Showing our lab members that rejection is the rule, rather than the exception, will help them to navigate the turbulent waters of research, reduce the prevalence of the “impostor syndrome” [38], and boost their self-confidence. And because successes are not so common, they must be properly celebrated when they happen. Fortunately, this is a usual practice in many labs that also contributes to the establishment of fruitful personal and professional interactions between lab members.

## Rule 10: Promote the professional development of your lab members

There is no single way this rule can be put into practice, because it may vary markedly among fields, countries, cultures, and personal situations. However, getting informed and openly discussing with lab members the pros and cons of all possible career options can help to do so. PIs should also allow time and resources (whenever available) to allow those lab members wishing to continue with a career in science to get trained in critical aspects of this job, such as experimental design, statistical analyses, and scientific writing. In addition, PIs should facilitate that graduate students and postdocs develop their own network of contacts, something that can be fostered by attending scientific meetings, by conducting research stays in other labs, and by participating in networks of scientists and specialist groups within scientific societies. Finally, PIs should also allow graduate students and postdocs to supervise BSc and MSc theses, respectively, on their own or under PI cosupervision, and offer postdocs the possibility of cosupervising new PhD students. By doing so, graduate students and postdocs acquire key experience on how to supervise the work of students, a critical task in academia; students get another view and critical inputs that end up improving their training and work; and PIs can have more time to do other important day-to-day issues that are needed to run a research lab. Furthermore, this action also effectively contributes to fostering collaborations and personal relationships between lab members.

## Concluding remarks

Strong competition promoted by the scarcity of funding and positions will continue to characterize science in many countries over the coming years. However, its negative effects on the performance and health of researchers [4, 5, 13, 17, 33] and the prevalence of such effects among new PIs, graduate students, and postdocs [1, 17–19] call for a more humane and people-centered way of working within research labs.

The 10 rules presented here can be adapted and refined to the circumstances of each research lab and can go a long way in improving working conditions and minimizing the negative effects of current scientific practice around the world. Embracing these rules could also help to alleviate other major problems faced by science today that can be exacerbated by extreme competition and the need to “be the first,” such as publication of poor-quality data, reduced research standards, fraudulent behavior, and lack of reproducibility [11, 13–15, 39, 40]. For the rules presented here to work, as PIs, we should practice them on a daily basis. If we stay for long hours in the lab, work on the weekends, and spend our holidays doing fieldwork, attending conferences or catching up with the literature, how can we convince our lab members of the need to have a proper life–work balance?

The 10 rules described here may not work for everyone. There will always be scientists who prefer to sacrifice part of their personal lives or health to be as productive and successful as possible, even though working for long hours may not help them to do so. Some scientists work in countries where the institutional system and/or prevailing cultural practice make it very difficult to implement them. But even in these cases, the application of some of the rules presented (e.g., Rules 1–5 and 9) can alleviate many of the problems associated with unhealthy working habits. It is also important to remember that, in the end, our work is only part of our life, and that achieving a proper life–work balance will make us healthier, happier, and more productive in the long term [34, 35]. Reasons for this include a lower stress load, a higher capacity to concentrate on important tasks, and boosted energy, satisfaction, and motivation levels [33, 34].

As PIs, we have a major influence on how the people working with us will behave once they become PIs. Therefore, we must not only act with probity and apply sound scientific practices and decisions but do as much as we can to make our labs more humane, collaborative, and healthy. We must also use any influence we have beyond our own labs to change current scientific and institutional practices, something that potentially can benefit thousands of scientists worldwide.

To sum up, something that I always keep in mind as a marathon runner: a research career is like a long-distance race, in which running too fast in the first kilometers can lead to a drop-off of the race before reaching the finish line. Therefore, let’s help our teammates to enjoy (and finish) the race by promoting a healthy and less stressful working environment.
